# The Physiology and Pathology of the Peridentium

**Published:** 1899-04

**Authors:** W. C. Wardlaw


### The Physiology and Pathology of the Peridentium.

BY W. C. WARDLAW, D.D.S.

It is well for us to be exact in our adoption of technical terms, and in writing of the tissue which associates the roots of teeth with their alveoli, I prefer to designate it as "peridentium," rather than "pericementum," or "periostium." Why so? Because this term is distinctive, and more descriptive than either of the others. It means the fibrous tissue, surrounding a tooth, and fibrous tissue located elsewhere should not therefore be confounded with it. The other two terms are applicable, it is true, to a certain extent. "Pericentum," as its derivation implies, is the membrane surrounding and nourishing the cementum, and "periosteum" is the fibrous tissue covering superficies of all bone, sending prolongations into its substance--is the bone builder--but this particular tissue is a membrane, associated with cementum on the one side and bone upon the other, belonging to both, but to neither exclusively. We therefore give it the designation "peridentium," which is comprehensive and inapplicable elsewhere. With the elimination from our nomenclature of these two terms go also their objectionable companions, viz: "Pericementitis" and "periostitis," the latter, as limited to this anatomical locality, being a glaring misnomer.

Now, then, the peridentium is exceptionable, being as it were a double membrane, lying between the cementum and the bony alveolus, nourishing them both, and attaching one to the other. The studies of Black throw much light upon the nature of the membrane. He describes the distribution of its semi-elastic fibers as being peculiar. They are attached to the cementum and proceed transversely, not perpendicularly, but at acute angles, to their union with the alveolus, suspending the tooth thus in a kind of elastic jacket. The articulation, although permitting some motion of the tooth, is not what is known as a movable joint, but is a "gomphosis," or nail-like union. The tooth when pressed upon in any direction will yield a little, and return to its proper position when relieved.

This beautiful arrangement constitutes a resilient cushion, upon which is received the numerous and violent impulses experienced in mastication. Instead of being transmitted as sudden shocks, through the tooth to the bone, they are distributed and dissipated, and thus made tolerable. Think of the disagreeable jars and painful irritations we would be subject to, of the numerous cases of peridentitis from which we would suffer! Every day we meet with teeth uncomfortable and inflamed from having thrown upon them excessive duty in masticating, by reason of the loss of antagonizing teeth.

The great strength of this attachment is also remarkable. We often have a practical exemplification of it in our efforts to extract a cuspid. Sometimes it requires the putting forth of our most strenuous efforts to break up its attachment and dislodge it from its alveolus. There is here no retentive power acquired through the mechanical shape of the tooth, for the root is straight and conical, the most favorable shape possible for its dislodgement. The tooth is retained solely by the strength and intimacy of association of this membrane with it and the bone. Often fragments of process are brought away with the tooth. We always find the peridentium adhering to an extracted tooth, showing its very close union, and affording another argument for the term "peridentium," rather than "periosteum."

Does this membrane retain its vitality when severed from the economy? Can it be resuscitated? Can a dry tooth be induced to resume a vital relation with the system? Is "implantation" a rational procedure?

The peridentium is well supplied with blood vessels and nerves, entering at the apical space, traversing its substance and freely anastamosing with like organs from bone and cementum. It is the medium through which the tooth may be nourished and kept alive, even after the death of the pulp. If forcibly torn away from the cementum the magnifying glass would reveal minute red spots, ruptured blood vessels, just as in case of periosteum torn up from the bone. We may therefore call it a well-organized tissue.

Now, physiology clearly teaches that the more vascular an organ is the more blood it contains, the more promptly will it succumb when the source of nutrition is cut off. An organ of low vitality does not yield so readily to death, as is exemplified in cold-blooded animals, which have but a sluggish circulation. In the very low orders a part severed from the parent-being may take on new functions and become a new being. Observe, its existence is not interrupted, but only diverted. It is said that human hair may continue to grow after the death of the body. And it is maintained that vitality may lie dormant in seeds for years, and then be brought to manifestations of activity by being placed in favorable environments. I believe that is an admitted fact that grains of wheat which have remained enshrouded with Egyptian mummies for ages have sprouted and grown when planted. But are these instances analagous to a dry tooth?

But we deny that the shriveled peridentium upon a dried specimen can ever be aroused to vital energy and be induced to put forth functional activity sufficient to re-establish actual systemic relations. Whilst the organization of the peridentium is not of the highest grade, we have seen that it is not sufficiently low to render probable a resumption of severed vital activity. All analogy, anatomy and physiology say that however firmly and closely attached an implanted tooth may seem to be, still there can be no real union between the dead and the living.

Another interesting question suggests itself. Can peridentium be reproduced upon a spot from which it has been cut away? It may require an intimate knowledge of the histology of the tissue to answer this query. There is no doubt but that skin-tissue will re-cover extensive arrears of denuded surface. Bone-grafting, through the periosteum, too, is a proved fact. The process is accomplished by the shooting forth in outward directions of little radials or minute buds of tissus-fiber, which go out from the circumference in search of distant footholds. Sponge grafting (which I believe to be the "modus operandi" of the retention of an implanted tooth) is an illustration of how tissue extend themselves. Can a denuded spot of cementum be recovered in this way? If so, there might be a hope for the radical cure of pyorrhœa alveolaris. Such a thing, in my opinion, might be possible if the spot is very small, or if it were in the midst of peridentium, maintaining upon its other side a connection with the bone or, in other words, if the peridentium is simply lifted from the tooth but not disconnected from the adjacent bone. But where tartar or what not, has dissected away the connection between the peridentium and the bone, I do not believe the union can ever be restored, although Dr. Atkinson claims to have brought back lost gum-tissue to the original line of attachment.

And just here I would venture to take issue with some of the different modes of treatment of pyorrhoea alveolaris. They are irrational, in that they do just exactly the contrary to what they are designed to accomplish. The whole treatment is destructive in its operation. With instruments the parts are scraped, lacerated, torn and denuded, and with acids and caustics are further destroyed, with the avowed purpose of removing diseased tissue and exciting a new and healthy action of the parts. As far as an instrument can be thrust the search for calculus is carried, the surrounding attachments are broken up and a pocket for the accumulation and retention of foreign matter is made.

I admit that the removal of diseased tissue and calculus is essential, " a sine qua non," in the curative process, but I aver that it will certainly be inoperative, unless the environments are put in condition conducive to restoration. It is a physical impossibility to keep these pockets free from the presence of destructive irritants. Healing and union cannot take place. Then why make them? And why retain them? They but serve to extend and increase the disastrous trouble.

My theory is, do not attempt an impossible restoration of already lost parts, but endeavor to preserve what is still left. Cut away, lop off, destroy, remove with knife and cautic, arsenic and caustic potash, all of the loose gum-tissue and carious bone down to the health line. Make self-cleansing spaces. Of course we denude the tooth to this extent, but a half loaf is better than none. We see where denuded roots, as the palatine roots of superior molars and the lingual surfaces of inferior incisors continue to do good service for a long while, if they have no pocket for the rentention of food, etc.

Still another function of the peridentium is the sense of touch. It is the tactile organ for the tooth, through which is transmitted to the sensorium all external impressions. All of the discriminations as to the nature of food, its density and size, are made through this medium. The pulp has none of this sense of touch, and being enclosed inside of the tooth cannot transmit impressions made upon the enamel. It transmits painful sensations only when it is itself irritated.

We have here valuable aids in differential diagnosis. If the pulp is exposed or inflamed, a sudden touch of heat or cold causes pain in it. If it is dead, cold or heat will not affect it. Thermal shocks will not affect an inflamed peridentium, except slightly and gradually. A blow would not hurt an inflamed pulp. Pains about the eye, ear or cheek may be expected to proceed from a diseased pulp, and not from peridentitis. Still I would emphasize the fact that occasionally neuralgic pains do by reflex action proceed from exquisite sensitiveness of the cementum when but slightly exposed at the necks of the teeth. I have had patients where a touch of the finger-nail, for instance, on these spots, or a very acid condition of the saliva, would give neuralgia.

The cutting out and filling of the sensitive portion would immediately cure the trouble.

I had intended taking up some of the pathological conditions of the peridentium, but consider that you have been sufficiently bored for the present.

